# The Role of the Transcription Factor SIM2 in Prostate Cancer

**DOI:** 10.1371/journal.pone.0028837

**Published:** 2011-12-09

**Authors:** Bin Lu, John M. Asara, Martin G. Sanda, Mohamed S. Arredouani

**Affiliations:** 1 Department of Surgery, Beth Israel Deaconess Medical Center, Harvard Medical School, Boston, Massachusetts, United States of America; 2 Department of Medicine, Beth Israel Deaconess Medical Center, Harvard Medical School, Boston, Massachusetts, United States of America; University of Saarland Medical School, Germany

## Abstract

**Background:**

Recent reports have suggested a possible involvement of Single-minded homolog 2 (SIM2) in human solid cancers, including prostate cancer. However, the exact role of SIM2 in cancer in general, and in prostate cancer in particular, remains largely unknown. This study was designed to elucidate the role of SIM2 in prostate cancer using a shRNA-based approach in the PC3 prostate cancer cell line.

**Methods:**

Lentiviral shRNAs were used to inhibit SIM2 gene and protein levels in PC3 cells. Quantitative RT-PCR and branched DNA were performed to evaluate transcript expression. SIM2 protein expression level was measured by western blot. Profiling of gene expression spanning the whole genome, as well as polar metabolomics of several major metabolic pathways was performed to identify major pathway dysregulations.

**Results:**

SIM2 gene and protein products were significantly downregulated by lenti-shRNA in PC3 cell line. This low expression of SIM2 affected gene expression profile, revealing significant changes in major signaling pathways, networks and functions. In addition, major metabolic pathways were affected.

**Conclusion:**

Taken together, our results suggest an involvement of SIM2 in key traits of prostate tumor cell biology and might underlie a contribution of this transcription factor to prostate cancer onset and progression.

## Introduction

Single-minded homolog 2 (SIM2) gene is located on the human chromosome 21q22.2 and is a member of the basic helix-loop-helix PAS [per-Arnt-Sim] (bHLH-PAS) family of transcription factors [1], [2]. SIM2 was originally thought to contribute to Down's syndrome (DS) [3]. As a transcription factor (TF), murine SIM2 (mSIM2) mediates gene expression through CNS midline enhancer (CME) element with its dimerization partner ARNT via ARNT carboxy-terminus [4]. The transcription factor c-myb regulates SIM2 transcription in glioblastoma cells, and a nuclear localization signal (NLS) mediates nuclear localization of SIM2 [5].

A prior *in silico* bioinformatics approach using the Cancer Genome Anatomy Project (CGAP) database of the National Cancer Institute (NCI) identified SIM2 as associated with colon, pancreas and prostate carcinomas, while absent in the corresponding normal tissues [6]. Two different spliced isoforms of SIM2 transcript, SIM2-long (SIM2-l) and SIM2-short (SIM2-s), have been reported while their differential function in humans are not known yet [1]. SIM2-s was specifically expressed in early stages of colon cancer. Antisense inhibition of SIM2-s expression by antisense oligos caused growth inhibition and apoptosis in colon cancer cell line RKO and tumor growth in nude mice and also in pancreatic cancer cell line CAPAN-1 [7], [8]. Apoptosis was induced by SIM2-s inhibition in the RKO colon cancer cell line [9]. SIM2-s was also found to have tumor suppressive activity in breast cancer [10]. The invasion potential of glioblastoma was decreased significantly by SIM2s inhibition, consistent with a decrease in the expression of matrix metalloproteinase 2 at both mRNA and protein levels [11].

We have previously reported SIM2 as a potential biomarker and immunotherapy target for human prostate cancer [12]. Although SIM2-s expression (as measured by immunohistochemistry of prostatectomy specimens) has been associated with aggressive histopathology in prostate cancer, and overexpressing ectopic SIM2s enhanced survival in certain conditions in PC3AR+ cells [13], [14], the functional role of SIM2 gene in prostate cancer cell is largely unknown.

In this study we sought to elucidate the functional role of SIM2 in PCa using a gene silencing approach and characterization of molecular and functional changes by both gene expression profiling and metabolomic profiling.

## Materials and Methods

### Cell lines

The human PC3, LNCaP, VCaP and DU145 cell lines were purchased from the American Type Culture Collection (ATCC, Manassas, VA) and cultured as per ATCC's protocol. Benign PrEC cells, as described in Berger R et al, 2004, were kindly provided by Dr. W. Hahn at Dana-Farber Cancer Institute, Boston, MA.

### Transduction Particles

The pLKO.1-puro control lentiviral transduction particles, MISSION luciferase shRNA control lentiviral transduction particles and MISSION SIM2 shRNA lentiviral transduction particles were used to infect PC3 cell line (Sigma-Aldrich, Saint Louis, MO).

### Sample selection, RNA purification and reverse transcription

Ten benign and fourteen tumor radical prostatectomy tissue samples were obtained and total RNAs were processed as described in our previous work [12]. Cell line total RNA was isolated using TRIzol reagent (Invitrogen Corporation, Carlsbad, CA) according to the manufacturer's instructions. Purified RNA was quantified by NanoDrop ND-1000 Spectrophotometer (NanoDrop, Wilmington, DE). 500 ng of each cell total RNA was reverse transcribed into cDNA using oligo dT and superscript III reverse transcriptase (Invitrogen Corporation, Carlsbad, CA) under the manufacturer's instructions.

### Gene expression microarrays and analysis

250 ng total RNA was amplified using Ambion's MessageAmp II mRNA Amplification kit. Biotin-UTP was incorporated during the overnight in vitro transcription step according to the manufacturer's protocol. Gene expression was assessed using Affymetrix's (Santa Clara, CA) GeneChip U133 array (Plus 2.0 chip) arrays representing the whole human genome transcripts. 15 μg cRNA was fragmented and hybridized to arrays' according to the manufacturer's protocols as described previously [15]. The quality of scanned arrays images were determined on the basis of background values, percent present calls, scaling factors, and 3′-5′ ratio of β-actin and GAPDH using the BioConductor R packages. The signal value for each transcript was summarized using PM-only based signal modeling algorithm described in dchip. The PM only based modeling based algorithm yields less number of false positives as compared to the PM-MM model. In this way, the signal value corresponds to the absolute level of expression of a transcript [16]. These normalized and modeled signal values for each transcript were used for further high level bioinformatics analysis. During the calculation of model based expression signal values, array and probe outliers are interrogated and images spike are treated as signal outliers. The outlier detection was carried out using dchip outlier detection algorithm. A chip is considered as an outlier if the probe, single or array outlier percentage exceeds a default threshold of 5%. When comparing two groups of samples to identify genes enriched in a given phenotype, if 90% lower confidence bound (LCB) of the fold change (FC) between the two groups was above 1.2, the corresponding gene was considered to be differentially expressed. LCB is a stringent estimate of FC and has been shown to be the better ranking statistic [17] It has been suggested that a criterion of selecting genes that have a LCB above 1.2 most likely corresponds to genes with an “actual” fold change of at least 2 in gene expression [18]. Data were extracted from CEL files and normalized using RMAexpress (http://rmaexpress.bmbolstad.com/). Data were analyzed using MeV software (http://www.tm4.org/mev/).

### Cell signaling pathway analysis

The Ingenuity Pathways Analysis (Ingenuity Systems®, http://www.ingenuity.com) applications were used to generate networks and assess statistically relevant biofunctions, canonical pathways and networks associated with the differentially expressed gene profiles extracted from the transcriptome data.

### Branched DNA and quantitative Real-Time PCR (qRT-PCR)

Branched DNA was performed to evaluate the SIM2s and SIM2L gene expression in the human prostate total RNA samples and normalized by 2 control genes ALSA1 and HPRT (QuantiGene 2.0 Reagent System, Affymetrix Inc, Fremont, CA). For quantitative RT-PCR, 1 μl cDNA was used for each well RT-PCR reactions. Samples were performed in triplicates. Taqman universal PCR Master Mix (Applied Biosystems, Foster City, CA) was used for two-step real-time RT-PCR analysis on Applied Biosystems 7900HT Prism instrument. Taqman real time PCR primers for GAPDH (4310884E) and SIM2L (hs00231925_m1) were purchased from Applied Biosystems (Foster City, CA). Taqman real time PCR primers for SIM2s were designed by our group and purchased from Biosearch Technologies (Novato, CA). SIM2s forward primer: 5′-gtgccaagct acgaaggtg-3′; SIM2s reverse primer: 5′-acttagaagcagaaagagggcaag-3′; probe: TCAGGTCTGCTCGTGGGGAAGGTG. Expression value of SIM2s or SIM2L in a given sample was normalized to the corresponding expression of GAPDH. The 2^–ΔΔCt^ method was used to calculate relative expression of SIM2 gene as described previously [19], [20].

### Lentiviral transduction and stable cell line selection

1.6 X 10^4^ PC3 cells were plated in 96 well plate and incubated for 20 hours. Medium was removed and 110 ul of fresh medium containing hexadimethrine bromide to a final concentration of 8 ug/ml were added. Lentiviral particles were added to appropriate wells at 5 MOI (multiplicity of Infection) and incubated overnight. Fresh medium was then added and cells cultured for 2 days, followed by a 10–12 days culture with puromycin (2 ng/ml) added every 3 days.

Transient transduction was achieved over a 3-day incubation.

### Western blot

Cells were washed twice with PBS twice before they were harvested by scraping. Cell lysates were prepared in cell lysis buffer (50 mmol/L Tris-HCl pH 8.0, 20 mM EDTA, 1% SDS, and 100 mM NaCl) containing an enzyme inhibitor mixture tablet (Roche Molecular Biochemicals, Indianapolis, IN) and PMSF (Sigma-Aldrich, Saint Louis, MO). Protein concentration was determined using BCA protein assay kit (Thermo Scientific, Rockford, IL). A total of 20–50 µg of protein extract was fractionated by SDS-PAGE and transferred to a polyvinylidene difluoride membrane (Immobilon-P; Millipore). The membrane was blocked with TBS-T (0.1% Tween 20 in PBS) containing 3% dry milk and incubated with SIM2s primary antibody (Santa Cruz, sc-8715, isoform NM_009586) overnight at 4°C. After three washes with TBS-T, the membrane was incubated with HRP-conjugated secondary Ab for 1 h and then washed with 0.05% Tween 20 in PBS. The immune complexes were detected by ECL methods (Thermo Scientific, Rockford, IL).

### Metabolite profiling using Targeted Liquid-Chromatography Tandem Mass Spectrometry (LC/MS/MS)

10^6^ cells exponentially growing in basal media with dialyzed serum were harvested in 3 mL 80% v/v HPLC grade methanol at dry ice temperatures. Fresh media was added 24 hours and 2 hours prior to the extraction. Insoluble material in lysates was centrifuged at 4000 RPM for 15 minutes and the resulting supernatant (metabolite content) was evaporated using a refrigerated SpeedVac to a pellet. Samples were re-suspended using 20μL HPLC grade water for mass spectrometry analysis. 10μL were injected and analyzed using a 5500 QTRAP triple quadrupole mass spectrometer (AB/Sciex) coupled to a Prominence UFLC HPLC system (Shimadzu ) via selected reaction monitoring (SRM) of a total of 255 endogenous water soluble metabolites for steady-state analyses of samples. Some metabolites were targeted in both positive and negative ion mode for a total of 298 SRM transitions. ESI voltage was +4900V in positive ion mode and –4500V in negative ion mode. The dwell time was 5 ms per SRM transition and the total cycle time was 2.09 seconds. Approximately 8–10 data points were acquired per detected metabolite. Samples were delivered to the MS via normal phase chromatography using a 2.0 mm i.d x 15 cm Luna NH2 HILIC column (Phenomenex) at 285 μL/min. Gradients were run starting from 85% buffer B (HPLC grade acetonitrile) to 42% B from 0–5 minutes; 42% B to 0% B from 5–16 minutes; 0% B was held from 16–24 minutes; 0% B to 85% B from 24–25 minutes; 85% B was held for 7 minutes to re-equilibrate the column. Buffer A was comprised of 20 mM ammonium hydroxide/20 mM ammonium acetate (pH = 9.0) in 95:5 water:acetonitrile. Peak areas from the total ion current for each metabolite SRM transition were integrated using MultiQuant v1.1 software (AB/Sciex).

Measurements were performed in triplicates and data were normalized per cell number. Only metabolites that were determined in all 6 samples were kept and analyzed using MetaboAnalyst [21],[22].

### Statistical analysis

Gene expression array data were analyzed as described under Materials and Methods. Based upon our earlier work [12], we tested for SIM2 upregulation in tumors versus controls with a one-sided t-test and compared against a p-value threshold of 0.05.

#### Quantitative Real-Time PCR (qRT-PCR)

Validation of differentially expressed genes was performed by qRT-PCR. 200 ng of high quality RNA samples were reverse transcribed to first strand cDNA and 1 μl cDNA was used for each well RT-PCR reaction. Samples were performed in triplicates. SYBR Green PCR Master Mix (Applied Biosystems, Foster City, CA) was used for two-step real-time RT-PCR analysis on Applied Biosystems 7900HT Prism instrument. PCR primers' sequences for targeted genes are shown in Table S3. The sequences for GAPDH: GAPDH-F (5′-TGCACCACCAACTGCTTAGC -3′) and GAPDH-R (5′-GGCATGGACTGTGGTCATGAG -3′). Expression value of the targeted gene in a given sample was normalized to the corresponding expression of GAPDH. The 2^–ΔΔCt^ method was used to calculate relative expression of the targeted genes.

## Results

### SIM2 gene is differentially expressed in prostate normal and cancer prostatectomy and cell lines

**Figure 1:**
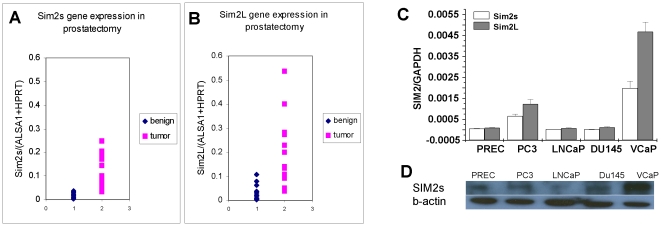
SIM2 expression in human prostatectomy, prostate normal and cancer cell lines. Quantitative Expression of SIM2 short isoform (A) and SIM2 long isoform (B) were evaluated by branched DNA technique in 10 normal and 14 human cancer prostatectomy specimens. Data were quantified using ALSA1 and HPRT as the normalizers. (C) Quantification of SIM2 short and long isoforms' expression in human prostate normal and cancer cell lines by real time RT-PCR. Data were quantified by the ΔΔC_T_ method and normalized to GAPDH. Column in white represents SIM2 short isoform and column in gray represents SIM2 long isoform. (D) Western blot were performed in prostate normal and cancer cell lines for SIM2s.

We have evaluated SIM2 gene expression in a total of 24 normal and tumor prostatectomy samples shown in table 1. Because SIM2 gene exists in two isoforms, SIM2 short (SIM2s) and SIM2 long (SIM2L), we confirmed the expression of both isoforms in RNA extracted from prostatectomy using branched DNA technique (Fig. 1A & 1B). SIM2s and SIM2L showed significant overexpression in tumor samples when compared to benign samples, with p < 0.000003 and p < 0.00005, respectively. However, the ratio of SIM2s to SIM2L expression was no difference between benign and tumor (table 1, T-test with p  =  0.85). The SIM2s and SIM2L expression were 7.03 and 6.95 times higher respectively in the tumors comparing the means of the two groups after a log adjustment to assure normality and constant variance within each group. Expression of SIM2s and SIM2L was also evaluated in four human prostate cancer cell lines, PC3, LNCaP, DU145 and VCaP, and in the normal prostate epithelial cell line PrEC. Both SIM2s and SIM2L isoforms were highly expressed in VCaP cells, while there was a moderate expression level in PC3 cells and very low expression in DU145, LNCaP, and PrEC cells (Fig. 1C). Because there are only a few available antibodies to SIM2, we have only been able to clearly identify the short isoform of SIM2 (SIM2s) in cellular protein extracts by western blot. This scarcity of antibodies complicated our task of studying the function of SIM2 long isoform. The SIM2s protein expression level was consistent with its gene expression in prostate normal and cancer cell lines. (Fig. 1D).

**Table 1:** Clinical information and SIM2 gene expression of 10 normal and 14 tumor prostatectomy evaluated by branched DNA technique.*

Case number	Tumor = 1 normal = 0	Gleason score	PSA level (ng/ml)	Sim2s mRNA expression **	Sim2L mRNA expression **	Sim2s/Sim2L ratio
315	0	N/A	7.6	0.0025	0.0024	1.041667
318	0	N/A	8.4	0.0057	0.008	0.7125
322	0	N/A	6.5	0.0182	0.0197	0.923858
334	0	N/A	5	0.0368	0.0645	0.570543
91	0	N/A	5	0.0215	0.0321	0.669782
20	0	N/A	7.2	0.01	0.0233	0.429185
149	0	N/A	7.1	0.0045	0.0057	0.789474
516	0	N/A	13.5	0.029	0.0379	0.765172
524	0	N/A	11.5	0.0338	0.0805	0.419876
544	0	N/A	7.8	0.0241	0.1094	0.220293
411	1	3+4 = 7	0.4	0.1449	0.2734	0.529993
417	1	3+4 = 7	4.2	0.2041	0.2281	0.894783
471	1	3+4 = 7	26.7	0.0414	0.0375	1.104
474	1	3+3 = 6	5.2	0.1711	0.2817	0.607384
478	1	3+4 = 7	4.4	0.0543	0.0921	0.589577
482	1	3+4 = 7	8.3	0.0513	0.1066	0.481238
523	1	3+4 = 7	8.7	0.2476	0.3987	0.621018
539	1	3+3 = 6	0.7	0.1956	0.5371	0.364178
545	1	3+3 = 6	3	0.1005	0.1996	0.503507
547	1	3+3 = 6	0.4	0.0824	0.134	0.614925
548	1	4+3 = 7	5.8	0.0606	0.1403	0.431932
303	1	N/A	6.4	0.0631	0.1084	0.582103
14	1	N/A	1.6	0.1812	0.2005	0.903741
125	1	4+3 = 7	18	0.0333	0.0494	0.674089

*Prostatectomy samples were from Hershey Tissue Bank at Beth Israel Deaconess Medical Center, Boston.

**mRNA expression values were normalized by ALSA1 and HPRT genes by branched DNA technique.

### Silencing SIM2 expression in PC3 cells

**Figure 2:**
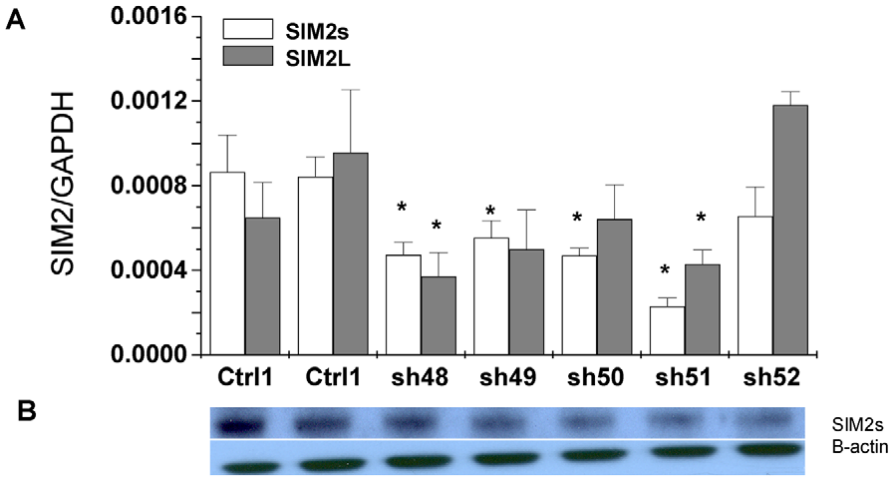
SIM2 expression in PC3 cells is downregulated by shRNA. Real time RT-PCR was performed in triplicates (A) and protein expression was evaluated by western blot (B). Control 1: Luciferase shRNA vector, Control 2: PLKO vector. sh48, sh49, sh50, sh50, sh51 and sh52: vectors expressing shRNAs targeting SIM2 gene at different sites. Column with “*” represents significant downregulation of SIM2 gene expression by shRNA comparing to both of control 1 and control 2 (P < 0.05).

To achieve the highest downregulation of SIM2 expression using lentiviral shRNA, we have selected the PC3 cell line as a model. PC3 cells were transduced with five different SIM2 shRNA expression vectors, four of which (shRNA48, shRNA49, shRNA50 and shRNA51) showed significant inhibitory effect compared to control shRNAs. Over 80% silencing of gene expression was achieved using shRNA51 (Fig. 2A&B). Two control cell lines were generated using either a vector stably expressing shRNA targeting luciferase or an empty vector. A similar inhibitory pattern was observed for SIM2L gene expression in these stably infected PC3 cell lines. Similarly, efficient transient silencing of SIM2S and SIM2L was achieved in PC3 (Figure S1).

### Impact of SIM2 silencing on gene expression profile in PC3 cells

Despite its suspected role in cancer, very little is known about the contribution of the transcription factor SIM2 to the regulation of gene expression [23]. We therefore examined the effects of downregulation of SIM2 in prostate cancer cells. To this end, the shRNA which yielded the highest silencing rate of SIM2, i.e. shRNA51, was selected. PC3 cells treated with shRNA51 were compared to a control shRNA (shRNAc).

**Figure 3:**
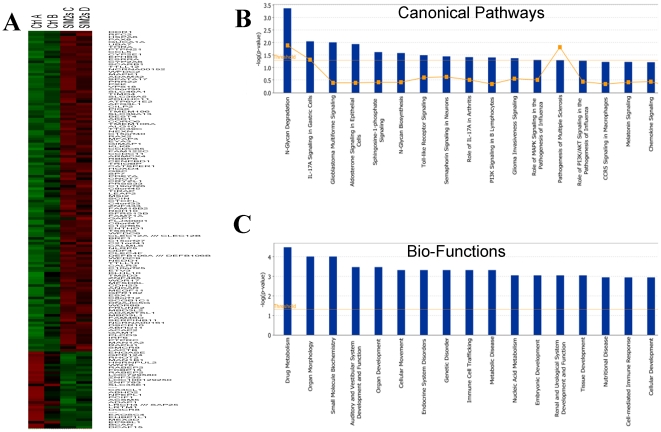
Heat map and cell signaling analysis for the dysregulated genes in SIM2^low^ comparing to control PC3 cells. A. Control A: PC3 luciferase shRNA; Control B: PC3 PLKO vector; SIM2 C: SIM2 sh48; SIM2 D: SIM2 sh51. Gene expressions were either up or down greater than 2 fold in the SIM2^low^ were listed. B. Top dysregulated signaling pathways in SIM2^low^ PC3 cells. C. Top dysregulated cell functions in SIM2^low^ PC3 cells.

**Figure 4:**
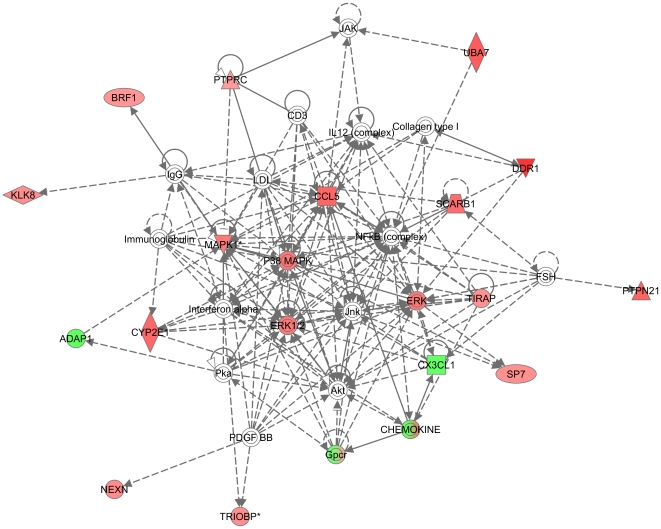
Top one network dysregulated in SIM2^low^. This network contained 16 focus genes with a score of 29. Different shapes of the node represent different groups of the focus genes. The intensity of the node color indicated the degree of the up (red) and down (green) gene expression level. The top functions of this network are cellular movement, immune cell trafficking, organismal injury and abnormalities.

Gene expression profiles of PC3 SIM2^low^ and control PC3 cell lines were evaluated using Affymetrix GeneChip U133 array (Plus 2.0 chip) consisting of >52,000 transcripts from whole human genome transcripts. Figure 1 is a heat map showing the gene dysregulation after knocking down the expression of SIM2 in PC3 cells. The expression of a large number of transcripts exhibited a change of at least 2-fold (Figure 3A and Table S4). Pathway analysis revealed that many highly differentially expressed transcripts represent genes that belong to known signaling pathways, such as the PTEN and PI3K/AKT signaling pathways (Figure 3B), whose involvement in tumorigenesis is well documented [24], [25]. Specific genes involved in each signaling pathway are shown in Table S1. Among those genes, CCL5, MAPK1, P38, DDR1 and ERK played a central role in the pathway network (Figure 4). The genes in this network have been involved in cell death, metabolism, cellular development, and tumor antigen presentation. More genes involved in the highest score networks are shown in Table S2. Further analysis showed that a number of important biological functions are dysregulated following SIM2 silencing (Figure 3C). Interestingly, several cell functions related to metabolism, such as drug metabolism and metabolic disease, are among the top ranked functions.

**Figure 5:**
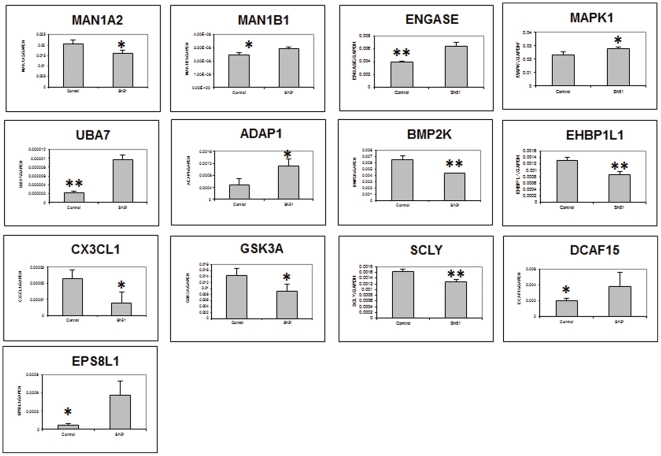
Validation of selected genes from stable transfectant by qRT-PCR. qRT-PCR validation of mRNA expression levels of individual genes was performed by two-step real-time RT-PCR analysis on Applied Biosystems 7900HT Prism instrument. *, P<.05; **, P<.01; ***, P<.001. Measurements were performed in triplicates and data presented as Mean ± SD.

**Figure 6:**
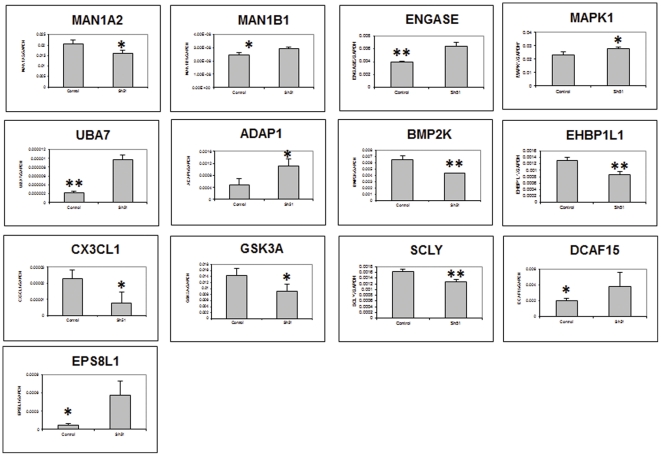
Validation of selected genes from transient transfectant by qRT-PCR. qRT-PCR validation of mRNA expression levels of individual genes was performed by two-step real-time RT-PCR analysis on Applied Biosystems 7900HT Prism instrument. *, P<.05; **, P<.01; ***, P<.001. Measurements were performed in triplicates and data presented as Mean ± SD.

Validation by RT-PCR of a group of differentially expressed genes (Table S3) partially confirmed our in silico analysis of stable and transient transfectant PC3 cells (Figures 5 & 6).

### PC3 SIM2^low^ cells showed major alterations in their metabolic profile

**Figure 7:**
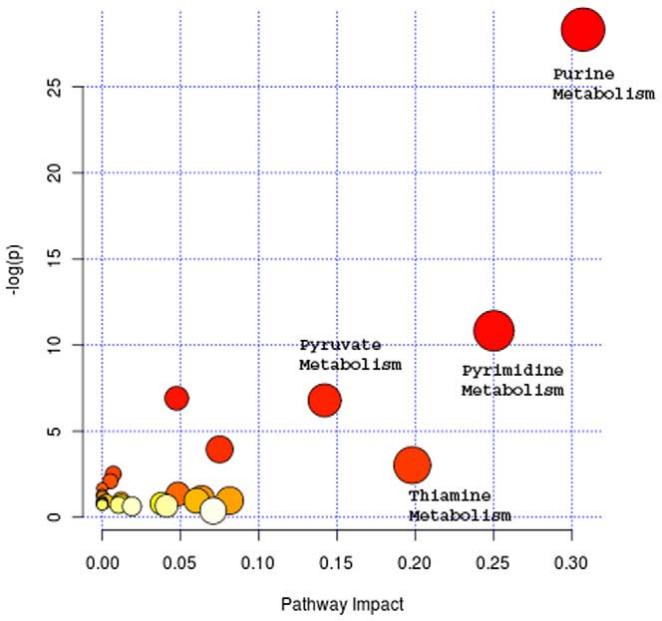
PC3 SIM2-shRNA cells showed major alterations in purine and pyrimidine metabolism pathways. Metabolites were extracted from SIM2^low^ and normal PC3 cells using methanol and the abundance of 239 metabolites were measured using targeted LC/MS/MS. Data were analyzed using MetaboAnalyst software. The metabolic pathways arranged according to the scores from enrichment analysis (y axis) and from topology analysis (x axis). Triplicate measurements were performed.

We sought to determine whether gene expression changes result in significant shifts in metabolic pathways in PC3 SIM2^low^ cells. This was addressed by measuring 255 polar metabolites using targeted mass spectrometry (LC/MS/MS). Comparison of the metabolic profile of control cells to shRNA-SIM2-treated cells showed significant changes in several metabolic pathways and the production of 39 metabolites (Tables 2&3). The purine metabolism pathway was the top one dysregulated pathway with 11 metabolites significantly up- or down-regulated levels out of total 92 metabolites in this pathway in SIM2 silencing PC3 cells. Pyrimidine metabolism pathway listed as the second dysregulated pathway with 6 out of total of 60 metabolites with significant changed levels (Table 2, Fig. 7). The significant alterations to the nucleic acid metabolism may indicate its important role in the prostate cancer development.

**Table 2:** Top dysregulated metabolic pathways in Sim2^low^ PC3 cells.

Pathway Name	Total number of Metabolites	Hits
Purine Metabolism	92	11
Pyrimidine metabolism	60	6
Glycolysis or Gluconeogenesis	31	3
Thiamine metabolism	24	2
Pyruvate metabolism	32	2

Metabolites were measured using mass spectrometry and data were analyzed using MetaboAnalyst software.

**Table 3:** List of dysregulated metabolites in Sim2^low^ PC3 cells.

Metabolite	P value*
UTP	0.00021736
CTP	0.000266674
Thiamine-phosphate	0.00027101
CDP	0.00027427
ATP	0.00032043
GDP	0.00047402
2-ketohaxanoic acid	0.00099085
dGTP	0.0011366
Adenosine	0.0025014
allantoate	0.0031712
4-Pyridoxic acid	0.0032341
Hydroxyphenylacetic acid	0.004154
IMP	0.0046357
Inosine	0.0052766
xanthosine	0.0056235
N-carbamoyl-L-aspartate	0.0057818
CDP-choline	0.0067616
lactate	0.007935
GTP	0.0084853
dCTP	0.011521
dATP	0.011737
Geranyl-PP	0.014616
Thiamine pyrophosphate	0.017058
dihydroxy-acetone-phosphate	0.017355
AMP	0.018114
Sn-glycerol-3-phosphate	0.019883
3-phosphoglycerate	0.021186
UDP	0.023169
dAMP	0.023309
Guanine	0.025068
Glucose-6-phosphate	0.027664
Fructose-6-phosphate	0.035608
Hexose-phosphate	0.036721
dTMP	0.044375
Guanosine 5-diphosphate, 3-diphosphate	0.046342
2-Isopropylmalic acid	0.047328
GMP	0.04827
hydroxyproline	0.049448

Metabolites were quantitated using mass spectrometry and data were analyzed using MetaboAnalyst software.

*P<0.05.

## Discussion

In our previous biomarker identification efforts, we have identified SIM2 as a potential biomarker for PCa. Thanks to its overexpression in prostate tumors and its highly restricted expression in humans, we proposed to use SIM2 as an immunotherapy target and were able to identify 5 HLA-A2.1, SIM2-derived immunogenic epitopes [12]. In the present study we attempted to characterize the role of SIM2 in prostate cancer using a short hairpin RNA-induced gene silencing approach in PC3 cells as a model. We focused on profiling both the transcriptome and metabolome in SIM2^low^ and normal PC3 cells, and evaluated the impact of SIM2 silencing on cell signaling and function.

The SIM2s isoform has been reported to be expressed in colon, pancreas, and prostate tumors while absent in the corresponding benign tissues [8]. We found that SIM2 genes are detectable in all these prostate cancer cells by real time PCR. However the expression levels in DU145 and LNCaP are relatively lower than other prostate cancer cells while PC3 cells express moderate level of SIM2 genes which are consistent with other report [14].

The whole spectrum of regulation of gene expression by the transcription factor SIM2 is still poorly defined. The level of regulation could be reflected by the differential expression of about 200 genes as revealed through gene expression profiling of PC3 SIM2^low^ cells. Other groups have reported specific genes that are regulated by SIM2. The bHLH/PAS transcription factor single minded 2s was reported to promote mammary gland lactogenic differentiation by regulation of Csn2 expression [26]. SIM2 regulates the expression of MMP-2 and TIMP-2, which drive its role in glioblastoma cells [11]. SIM2s represses BNIP3, a pro-apoptotic gene, through its hypoxic response element in PC3 cells [14]. Our gene expression profile in PC3 SIM2^low^ cells showed significant change in PTEN, PI3K/AKT and Toll-like receptor (TLR) signaling pathways which are involved largely in the tumor progression. PTEN negatively controls the PI3K signaling pathway for cell growth and survival by dephosphorylating the 3 position of phosphoinositides [24], [25]. TLR regulates cell proliferation and survival and central signaling molecules mitogen-activated protein kinase (MAPK) and PI3K play key roles [27]. Our data show that inhibition of Sim2 gene in PC3 cells affects expression of several genes encoding proteins that are organized in a network around p38MAPK. These proteins, which include CCL5, MAPKs, ERK and DDR1 (Figure 4), have been reported to be involved in tumor development. The chemokine CCL5 has been reported to be expressed by prostate cells and affect their growth and survival. Following activation of MAPKs p38 and ERK1/2 in LNCaP cells, the expression of CCL5 increases, resulting in enhanced cell proliferation [28], [29]. PC3 cell proliferation and invasion were also significantly suppressed after DDR1 knockdown by siRNA [30], [31].

Our RT-PCR data revealed discrepancies between transient and stable silencing of SIM2 in PC3 cells. This may be a result of 1) the presence of two isoforms of SIM2 that are silenced to different extents in both setups, or 2) SIM2 may regulate gene expression of other genes either directly or indirectly.

Function analysis also revealed that three functions related to cell metabolism had been dysregulated in the PC3 SIM2^low^ cells. This suggested that SIM2 might have metabolic consequences. We have evaluated the production by PC3 cells of 255 metabolites that encompass a large number of human metabolic pathways. Of these, data were obtained for 239 metabolites. Our analysis revealed significant changes in metabolites that constitute key pathways, such as the purine and pyrimidine pathways.

Suppression of SIM2 short isoform (SIM2s) by antisense oligonucleotides reduced tumor growth in colon cancer cells and induced CAPAN-1 pancreatic cell death through apoptosis [7], [8], [9]. SIM2s was also reported to be an aggressive prostate cancer biomarker since SIM2s protein was associated with increased preoperative serum prostate specific antigen (PSA), high histological grade, invasive tumor growth and increased tumor cell proliferation [13]. A recent study showed that SIIM2s may attenuate cell death processes through BNIP3 repression in PC3AR+ cells. However, knockdown of SIM2s in breast cancer MCF-7 cells increased tumorigenesis and thus showed tumor suppressor activity [32], [33]. Most of the previous studies focused on the SIM2s by either intruding or knockdown of SIM2s, we are lacking of the data clarifying the functional role of SIM2 protein including both of its isoforms. Our study reported a combined role of both isoforms of the SIM2 implicated in the prostate cancer cell. Distinguishing the roles of SIM2s and SIM2L may have more profound meaning to understand the functional role of SIM2 in prostate cancer progression, which is our next step to uncover more significance of this gene.

## Supporting Information

Figure S1 Transient silencing of SIM2s and SIM2L expression in PC3 cells. PC3 cells were transduced with either a control (Ctrl) or shRNA51 (sh51) and cultured in the presence of puromycin for 3 days. Real time RT-PCR was performed in triplicates to evaluate gene expression of SIM2 s (Upper Panel) and SIM2L (Lower Panel).
(TIF)

Table S1 The top Dysregulated Signaling Pathways in SIM2^low^ cells. Top dysregulated canonical pathways were identified through analysis of differentially expressed gene data, using Ingenuity Pathway Analysis package.
(DOC)

Table S2 The Molecules in the Highest Score Networks in SIM2^low^ cells. Data representing differentially expressed genes were submitted to Ingenuity Pathway Analysis package and high score networks were identified.
(DOC)

Table S3 List of primers used for RT-PCR quantitation of expression of of selected genes. The primers were designed using Pimer3 program: http://frodo.wi.mit.edu/primer3/.
(DOC)

Table S4 Gene expressions that were either up- or down-regulated greater than 2 fold in the SIM2^low^.
(XLSX)
