# Supplementary figures and images for: The Role of the Transcription Factor SIM2 in Prostate Cancer
Source: PLoS One. 2011 Dec 9;6(12):e28837. doi: 10.1371/journal.pone.0028837 (PMC3235151; doi:10.1371/journal.pone.0028837)

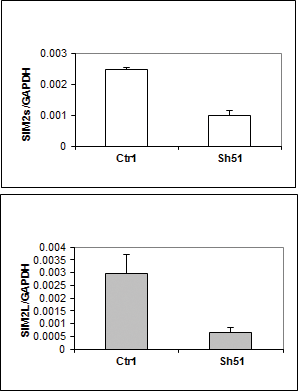

Supplement: Figure S1 — Transient silencing of SIM2s and SIM2L expression in PC3 cells. PC3 cells were transduced with either a control (Ctrl) or shRNA51 (sh51) and cultured in the presence of puromycin for 3 days. Real time RT-PCR was performed in triplicates to evaluate gene expression of SIM2 s (Upper Panel) and SIM2L (Lower Panel). (TIF) [file pone.0028837.s001.tif]
