# Supplementary material for: The Role of the Transcription Factor SIM2 in Prostate Cancer
Source: PLoS One. 2011 Dec 9;6(12):e28837. doi: 10.1371/journal.pone.0028837 (PMC3235151; doi:10.1371/journal.pone.0028837)
Supplement: Table S1 — The top Dysregulated Signaling Pathways in SIM2low cells. Top dysregulated canonical pathways were identified through analysis of differentially expressed gene data, using Ingenuity Pathway Analysis package. (DOC) [file pone.0028837.s002.doc]

| Table S1. The top Dysregulated Signaling Pathways in SIM2^low^ cells | | | |
| --- | --- | --- | --- |
|  | | | |
| **Ingenuity Canonical Pathways** | **-log(p-value)** | **Ratio** | **Molecules** |
| N-Glycan Degradation | 3.37 | 0.115 | MAN1A2, MAN1B1, ENGASE |
| IL-17A Signaling in Gastric Cells | 2.05 | 0.08 | MAPK1, CCL5 |
| Glioblastoma Multiforme Signaling | 2.01 | 0.0244 | PLCD3, MAPK1, RHOT2, WNT6 |
| Aldosterone Signaling in Epithelial Cells | 1.94 | 0.0235 | PLCD3, DNAJC5G, MAPK1, HSPA6 |
| Sphingosine-1-phosphate Signaling | 1.62 | 0.0252 | PLCD3, MAPK1, RHOT2 |
| N-Glycan Biosynthesis | 1.58 | 0.0247 | MAN1A2, MAN1B1 |
| Toll-like Receptor Signaling | 1.5 | 0.0364 | MAPK1, TIRAP |
| Semaphorin Signaling in Neurons | 1.45 | 0.0385 | MAPK1, RHOT2 |
| Role of IL-17A in Arthritis | 1.42 | 0.0317 | MAPK1, CCL5 |
| PI3K Signaling in B Lymphocytes | 1.41 | 0.021 | PTPRC, PLCD3, MAPK1 |
| Glioma Invasiveness Signaling | 1.38 | 0.0333 | MAPK1, RHOT2 |
| Role of MAPK Signaling in the Pathogenesis of Influenza | 1.31 | 0.0308 | MAPK1, CCL5 |
| Pathogenesis of Multiple Sclerosis | 1.3 | 0.111 | CCL5 |
| Role of PI3K/AKT Signaling in the Pathogenesis of Influenza | 1.27 | 0.027 | MAPK1, CCL5 |
| CCR5 Signaling in Macrophages | 1.23 | 0.0213 | MAPK1, CCL5 |
| Melatonin Signaling | 1.23 | 0.026 | PLCD3, MAPK1 |
| Chemokine Signaling | 1.22 | 0.027 | MAPK1, CCL5 |
