# Supplementary material for: The Role of the Transcription Factor SIM2 in Prostate Cancer
Source: PLoS One. 2011 Dec 9;6(12):e28837. doi: 10.1371/journal.pone.0028837 (PMC3235151; doi:10.1371/journal.pone.0028837)
Supplement: Table S2 — The Molecules in the Highest Score Networks in SIM2low cells. Data representing differentially expressed genes were submitted to Ingenuity Pathway Analysis package and high score networks were identified. (DOC) [file pone.0028837.s003.doc]

| Table S2. The Molecules in the Highest Score Networks in SIM2^low^ cells * | | | | |
| --- | --- | --- | --- | --- |
|  | | | | |
| **Rank** | **Molecules in Network** | **Score** | **Focus Genes** | **Top Functions** |
| 1 | ADAP1, BMP2K, C12ORF47, CORO1B, CX3CL1, CYLD, CYP2E1, DDR1, EPS8L1, GSK3A, IL17RC, MAPK1, PPP2R5B, PTPN21, SCARB1, SCLY, SYNRG, UBA7, ZNF335 | 41 | 20 | Organismal Injury and Abnormalities, Cell Death, Carbohydrate Metabolism |
| 2 | AP1M2, DDR1, MAN1B1, RABEP2, RCN3, RFC2, RIN3, TIMD4, WFDC2, WNT6 | 17 | 10 | Cellular Development, Cell Cycle, Cell-To-Cell Signaling and Interaction |
| 3 | BCAN, BMP2K, CACNG4, CILP2, EPHB3, FERMT1, GIMAP5, PAX8, RHOT2, TXRND3 | 17 | 10 | Endocrine System Disorders, Genetic Disorder, Metabolic Disease |
| 4 | CALML4, CASKIN2, CYP2A6, DCAF15, FKBP15, GUCA1A, PCIF1, RAB15, SUGP2, VPS18 | 16 | 10 | Cellular Movement, Hematological System Development and Function, Immune Cell Trafficking |
| 5 | CCL5, ESRRA, GPR124, GPR153, HIF1AN, HSPA6, THRA, ZDHHC11 | 13 | 8 | Antigen Presentation, Cellular Movement, Hematological System Development and Function |
| 6 | AFG3L1, EXOSC4, INO80B, SHQ1, TTLL12, UBE2D4, WDR48 | 11 | 7 | Cellular Function and Maintenance, Cardiac Arteriopathy, Cardiovascular Disease |
| * P < 0.002 | | | | |
