# Supplementary material for: The Role of the Transcription Factor SIM2 in Prostate Cancer
Source: PLoS One. 2011 Dec 9;6(12):e28837. doi: 10.1371/journal.pone.0028837 (PMC3235151; doi:10.1371/journal.pone.0028837)
Supplement: Table S3 — List of primers used for RT-PCR quantitation of expression of of selected genes. The primers were designed using Pimer3 program: http://frodo.wi.mit.edu/primer3/. (DOC) [file pone.0028837.s004.doc]

| Additional Primers information | |
| --- | --- |
| Name | Sequences 5’-> 3’ |
| PTPRC-f | ccccggactctttggataat |
| PTPRC-r | agggttgagttttgcattgg |
| PLCD-f | ctcccagaaccactctcagc |
| PLCD-r | gttgaagccattgttgagca |
| MAPK1-f | tgatcacacagggttcctga |
| MAPK-r | tggaaagatgggcctgttag |
| CCL5-f | cgctgtcatcctcattgcta |
| CCL5-r | acacacttggcggttctttc |
| DDR1-f | attttgatcctgccaagtgc |
| DDR1-r | ccaggtgcagtcgttgtaga |
| MAN1A2-f | tcctagatgggcaaagatgg |
| MAN1A2-r | cagccaattgcactgcttta |
| MAN1B1-f | gtggatcttgggtctgagga |
| MAN1B1-r | gcattagccgatttccaaaa |
| ENGASE-f | ggaaatgtggctgtgaggtt |
| ENGASE-r | gcttgtttctgcgttcaaca |
| RHOT1-f | atccagagagggagacacga |
| RHOT1-r | gctcatcaggtgacaaagca |
| WNT6-f | ggttatggaccctaccagca |
| WNT6-r | gtctcccgaatgtcctgttg |
| RFC2-f | gatcatcgagcccattcagt |
| RFC2-r | ccaaatcctgagaaggtgga |
| HSPA6-f | aggaggtggagaggatggtt |
| HSPA6-r | tgtcctcttcgggaatcttg |
| PAX8-f | tcaacctccctatggacagc |
| PAX8-r | ctgctgctgctctgtgagtc |
| GUCA1A-f | cagccagtacgtggaacaga |
| GUCA1A-r | ctgcagtcatggtggtatcg |
| EHBP1L1-f | ccagataagctggtggtggt |
| EHBP1L1-r | ggtccctgtagagggtcaca |
| MEX3D-f | cgcaagaaaagcgtcaacat |
| MEX3D-r | gcggatgatggagaagtgtt |
| EPS8L1-f | gacgtgctgcagaagatcaa |
| EPS8L1-r | aggtccagagctcgttttca |
| BCAN-f | ggactcaacgacaggaccat |
| BCAN-r | gcaggtgtaggacaggtggt |
| DCAF15-f | tgccaaatcctgtatgacca |
| DCAF15-r | tgtcagccacaaactccttg |
| ADAP1-f | gacagaacgagagggtgctc |
| ADAP1-r | gttacgggtgctgttgtcct |
| BMP2K-f | gcaacacagcagcaacagat |
| BMP2K-r | ttgagacggctgatgttgag |
| C12ORF47-f | gagattcacgcctggaaaac |
| C12ORF47-r | gacgaaaggaggaaatgcag |
| CORO1B-f | ttacctgggacagcaccttc |
| CORO1B-r | tggctatgacttcgtcgttg |
| CX3CL1-f | cccaaaactctcctctgctg |
| CX3CL1-r | cagcaggaccaagagacaca |
| CYLD-f | tgcccctcttgacagagact |
| CYLD-r | ggccacacttgctggttaat |
| CYP2E1-f | acccgagacaccattttcag |
| CYP2E1-r | tccagcacacactcgttttc |
| GSK3A-f | actccagtggcgagaagaaa |
| GSK3A-r | ttgaggacagcagtgtcagg |
| IL17RC-f | ctcagcccaggtacgagaag |
| IL17RC-r | cggtccagtcaggtttttgt |
| PPP2R5B-f | gagcgggctctgtatttctg |
| PPP2R5B-r | tccagcttgtaggaggctgt |
| PTPN21-f | gagaatgatgctggtgcaga |
| PTPN21-r | cattcagactgcgccactta |
| SCARB1-f | ctgtgggtgagatcatgtgg |
| SCARB1-r | gccagaagtcaaccttgctc |
| SCLY-f | aactcagggtttcagctcca |
| SCLY-r | cgacatgtgatgtgtgtcca |
| SYNERG-f | agccagtcatgggcattaac |
| SYNERG-r | aagcaggcaactcttggaaa |
| UBA7-f | cggtacttccctagcacagc |
| UBA7-r | gttctgtggacgcactctca |
| ZNF335-f | agctttgcctccaagaacaa |
| ZNF335-r | tgacttcctcccatcaggac |
| AP1M2-f | tgatcagccgcaactacaag |
| AP1M2-r | agtacaccagggaggcattg |
| RABEP2-f | ccaggtccagaactcagagc |
| RABEP2-r | agcccttggttttcctcatt |
| RCN3-f | gacttccgggatctgaacaa |
| RCN3-r | ccatagttggtggcctgact |
| RIN3-f | ccaggactcctactccacca |
| RIN3-r | tcttgtacagcttgcggttg |
| TIMD4-f | ccccagtgattcctggagta |
| TIMD4-r | ggagaaacgccacaaacaat |
| WFDC2-f | cggcttcaccctagtctcag |
| WFDC2-r | cctccttatcattgggcaga |
| The primers were designed using Pimer3 program: <http://frodo.wi.mit.edu/primer3/> | |
